# Globalization in clinical drug development for sickle cell disease

**DOI:** 10.1002/ajh.27525

**Published:** 2024-11-12

**Authors:** Enrico Costa, Russell E. Ware, Léon Tshilolo, Julie Makani, Hubert G. M. Leufkens, Lucio Luzzatto

**Affiliations:** ^1^ World Health Organization Collaborating Centre for Pharmaceutical Policy and Regulation, Division of Pharmacoepidemiology and Clinical Pharmacology Utrecht University Utrecht The Netherlands; ^2^ Division of Hematology and Global Health Center Cincinnati Children's Hospital Medical Center Cincinnati Ohio USA; ^3^ Institut de Recherche Biomédicale 1‐Health, CEFA‐Monkole Kinshasa Congo; ^4^ Department of Hematology and Blood Transfusion at Muhimbili University of Health and Allied Sciences, Dar es Salaam, Tanzania. Sickleinafrica, Dar‐es‐Salaam Tanzania and Imperial College London London UK; ^5^ Regulatory Science and Pharmaceutical Policy Utrecht University Utrecht The Netherlands; ^6^ Department of Hematology University of Florence Florence Italy

## 1 BACKGROUND

Globalization of clinical trials, defined operationally as conduct in the international arena, has grown over the past few decades. The pharmaceutical industry is expanding its activities not only in High‐Income countries but also in Low‐ and Middle‐Income countries (LMICs).^1^

For pharmaceutical companies, this shift can be associated with several benefits: a larger pool of potential participants, faster enrollment in trials, and substantial cost savings.^1^ At the same time, there may be advantages also for LMICs in terms of capacity building, gaining experience, and access to innovation.^2^

Drug development and access to medicines in LMICs is certainly a challenge for patients with sickle cell disease (SCD), a condition that is most highly prevalent in malaria‐endemic countries in the global South, but that, through the tragedy of the transatlantic slave trade and subsequent migrations, is also prominent in the global North.^3^

The prevalence of SCD outside Africa has accelerated the development of new medicinal products, enhanced by a conducive regulatory framework. The orphan drug legislation in the United States (US) and the European Union (EU) have provided pharmaceutical developers with special incentives (e.g., periods of market exclusivity) to counterbalance the limited market size. In addition, the US Food and Drug Administration (FDA) and the European Medicines Agency (EMA) have implemented special pathways to expedite the review and approval of treatments for serious and life‐threatening diseases. Accordingly, in both the US and the EU, treatments for SCD can be approved based on surrogate endpoints or more flexible evidence.^4^

To assess the trends and impact of globalization on the development of SCD drugs, we analyzed data from industry‐sponsored studies initiated in the time interval from 1 January 1990 through 30 June 2024 (see Appendix S1). In the study period, a total of 79 pharmaceutical active substances were tested in 156 clinical trials.

Overall, 56.4% of enrolling centers were in North America, 20.5% in Europe, 7.9% in Africa, 5.7% in Latin America, 9.1% in Asia and Middle East, and 0.4% in Australia. Temporal trends from the early 2000s to the last 5 years showed a relative decrease of enrolling centers in North America from 63.1% to 44.0%, and in Europe from 28.5% to 22.2%. By contrast, in African centers there was an increase from 0.5% to 13.2%, in Latin America from 1.1% to 9.0%, and in Asia and the Middle East from 5.7% to 11.4%. The number of Australian centers remained low over time.

Similar trends were mirrored in the drug development phases: for instance, enrolling centers in Africa increased from 2.8% in Phase 1 trials to 4.2% in Phase 2, and to 10.6% in Phase 3 and 11.9% in Phase 4 trials. Similar increases were observed in Latin America, Asia, and the Middle East (Appendix S2). When these trends are considered according to World Bank country classifications by income, we observe an expansion toward LMICs, where participating centers increased from 7.4% of all centers in Phase 1, to 15.1% in Phase 2, to 22.1% in Phase 3, to 41.3% in phase 4.

Against this background, we have detailed data from pivotal clinical trials conducted by pharmaceutical companies seeking regulatory approval for medicines for the treatment of SCD. As of December 2023, 10 trials have led to the licensing of 9 medicinal products for SCD in the US and the EU. These products range from small molecules such as hydroxyurea, L‐glutamine, voxelotor, and deferiprone, to biologicals (crizanlizumab), and most recently to two potentially curative gene therapies (Table 1).

**TABLE 1:** Pivotal clinical trials of medicinal products approved by the US Food and Drug Administration and the European Medicines Agency for Sickle Cell Disease.

	Hydroxyurea caps	Hydroxyurea tabs	L‐Glutamine	Crizanlizumab	Deferiprone	Lovotibeglogene autotemcel	Voxelotor tabs^a^	Voxelotor dispersible tabs^a^	Exagamglogene autotemcel	Crizanlizumab
Study reference	MSH (NCT00000586) Charache et al. N Engl J Med 1995	ESCORT HU (NCT02516579) de Montalembert, et al. Am J Hemat. 2021	(NCT01179217) Niihara et al. N Engl J Med 2018	SUSTAIN (NCT01895361) Ataga et al. N Engl J Med 2017	LA38‐0411(NCT02041299) Kwiatkowski, et al. Blood Adv. 2022	(NCT02140554)^b^ Kanter J et al. N Engl J Med. 2022	HOPE (NCT03036813) Vichinsky et al. N Engl J Med 2019	HOPE‐KIDS 1 (NCT02850406) Estepp JH et al. Pediatr Blood Cancer 2022	CLIMB SCD‐121^b^ (NCT03745287) Frangoul et al. N Engl J Med 2021	STAND^b^ (NCT03814746)
Drug licensing	US—1998 EU—national procedures	US—2017 EU—2007^c^	US—2017 EU—2019 (negative opinion)	US—2019 EU—2020^d^	US—2021	US—2023	US—2019 EU—2022	US—2021	EU—2023 US—2024	EU—2023 (revoked)
Study characteristics										
Study start	1992	2009	2010	2013	2014	2014	2016	2016	2018	2019
Design	Phase 3, multicenter, randomized (1:1), double‐blind, placebo‐controlled	Multicenter, open‐label, single‐arm, prospective, observational, cohort study	Phase 3, multicenter, randomized (2:1), double‐blind, placebo‐controlled	Phase 2, multicenter, randomized (1:1:1), double‐blind, placebo‐controlled	Phase 4 US/3b other countries, multicenter, randomized, open‐label (2:1), non‐inferiority, active‐controlled	Phase 1/2, multicenter, open‐label, single‐arm, single‐dose	Phase 3, multicenter, randomized (1:1:1), double‐blind, placebo‐controlled	Phase 2a, multicenter, open‐label, single and multiple dose	Phase 1/2/3, multicenter, open‐label, single‐arm, single‐dose	Phase 3, multicenter, randomized (1:1:1), double‐blind, placebo‐controlled
*N* patients enrolled	299	141^c^	230	198	230	50	274	45	45	240
Age	18–50 years	≥2 years	16–65 years	16–65 years	≥2 years	12–50 years	12–65 years	4–11 years	12–35 years	≥12 years
Eligible genotypes	βS/βS or βS/β0	βS/βS, HbSC, HbSD, βS/β0, βS/β+	βS/βS or βS/β0	βS/βS, HbSC, βS/β0, βS/β+, or other genotypic variants	βS/βS, HbSC, βS/β0, βS/β+, or other genotypic variants	βS/βS, βS/β0, or βS/β+	βS/βS, βS/β0, HbSC, or other genotypic variants	βS/βS, βS/β0	βS/βS or βS/β0	βS/βS, HbSC, βS/β0, βS/β+, or other genotypic variants
Centers										
*N* centers	21	65	31	56	33	11	62	18	17	61
Geographical distribution										
North America	*21 (100%)*	*0*	*31 (100%)*	*49 (87.5%)*	*9 (27.3%)*	11 (100%)	*30 (48.4%)*	*11 (61.1%)*	*10 (58.8%)*	*7 (11.5%)*
Latin America^(^ ^e^ ^)^	*0*	*9 (13.8%)*	*0*	*7 (12.5%)*	*5 (15.1%)*	*0*	1 (1.6%)	*0*	*0*	*15 (24.6%)*
Europe	*0*	*56 (86.2%)*	*0*	*0*	*4 (12.1%)*	*0*	*16 (25.8%)*	*4 (22.2%)*	*7 (41.2%)*	*29 (47.5%)*
Asia and Middle	*0*	*0*	*0*	*0*	*6 (18.2%)*	*0*	*7 (11.3%)*	*3 (16.7%)*	*0*	*8 (13.1%)*
Africa	*0*	*0*	*0*	*0*	*9 (27.3%)*	*0*	*8 (12.9%)*	*0*	*0*	*2 (3.3%)*

*Note*: The table reports data in chronological order of study starting from left to right. L‐Glutamine was approved in the US but withdrawn in the EU upon a negative opinion from the EMA. Lovo‐cel was approved only in the US, as the company closed its operations in Europe. Crizanlizumab, an anti‐P selectin antibody, is in limbo at the moment, still marketed in the US but withdrawn in the EU. This product was conditionally approved in the EU upon promising results of a Phase 2 SUSTAIN trial (NCT01895361); however, having failed to meet the primary endpoint in the confirmatory Phase 3 STAND trial (NCT03814746) it was revoked from the EU market. The reasons for the discrepancy between the two trials are not clear but may mean that P‐selectin is not an ideal target to block adhesion and vaso‐occlusion or refers to the patient selection.

Abbreviations: caps, capsules; tabs, tablets.

^a^ Sponsor's voluntary withdrawal on September 25, 2024.

^b^ Studies ongoing at the time of the analysis: patients enrolment refers to the study estimation.

^c^ In keeping with well‐established used procedures, the EU approval was based on literature data and data from registries. In the US, the FDA based its decision on data from 141 out of the 405 enrolled in the HU ESCORT study.

^d^ EMA conditional marketing authorization.

^e^ Latin America includes all countries from South and Central America.

In the face of a gradual but steady tendency of clinical trials to become more global, the purpose of this article is to consider some implications of this trend: these may become even more important in the future if, as we hope, the trend continues.

### 1.1 Clinical research issues

The rationale for testing a new medicine in different geographical settings is strong particularly for polygenic disorders, as other genes may come into play in various populations. However, there is a rationale also in the case of a monogenic disorder like SCD, because multiple factors in different environments can influence the clinical course of the disease, and therefore in principle the response to certain treatments may be different.

Hydroxyurea was approved in 1998 by the US FDA for “symptomatic SCA in adults,” and it has since become the standard of care for both adults and children with SCD.^4^ Initially, there was some reluctance to introduce hydroxyurea into geographical regions where SCD is most prevalent and, where conditions such as malnutrition, endemic malaria, and other infectious diseases coexist. This paradoxical reluctance was due in part to excessive and misplaced fear of treatment‐related side effects including drug toxicities, malignancies, sterility, and insufficient education of health workers. The need to provide evidence for the benefits of hydroxyurea in such settings led to the conception of the NOHARM (NCT01976416) and REACH (NCT01966731) trials. These two prospective studies have assessed the feasibility, safety, and efficacy of hydroxyurea in Africa: they have proved that—as was to be expected—hydroxyurea is both safe and efficacious for the management of patients with SCD, wherever they live.^5^, ^6^

The choice of study end‐points also merits attention. In the case of voxelotor, a significant increase in hemoglobin was demonstrated, while the annualized incidence of pain crisis was not decreased. This outcome was still interpreted as clinically significant since vaso‐occlusive events might have increased with higher blood viscosity due to improved hemoglobin levels. In Africa, where the severity of anemia in SCD is greater,^7^ the hemoglobin end‐point is probably more important than elsewhere. However, due to concerns about patient safety in current clinical trials, in September 2024 Pfizer withdrew voxelotor from all global markets while more investigation is underway.

Industry‐sponsored research encompasses a wide range of studies performed worldwide. It would be desirable for such studies, especially those that aim to further investigate efficacy and safety in the long term, to be conducted in LMICs. We also advocate that professionals working in Africa must themselves be involved in the design and conduct of these clinical trials.

### 1.2 Ethical issues

Several studies have reported that patients in LMICs may be more willing to accept possible risks associated with participation in clinical trials than those from countries where alternative treatments or supportive care are more readily available.^8^ According to the Declaration of Helsinki, vulnerable groups or communities involved in medical research should stand to benefit from the knowledge, practices, or interventions resulting from the research.^2^, ^8^

This is directly relevant to on‐study treatment but especially to post‐trial access. We found that among the trials listed in Table 1, only one study protocol, the STAND trial, stated clear measures to provide access to crizanlizumab to participants once the trial was finished. In others, there was no commitment to extend study treatment for enrolled participants. Trials that fail to provide such access are not in line with the Declaration of Helsinki and may cause the SCD patient community to have misgivings about participation in future trials.^2^

Even when post‐trial access is provided, very few patients would receive the new medicine. Furthermore, what about the majority of SCD patients not enrolled in the clinical trials and living in areas where the disease is most prevalent? In this respect, the notion of “reasonable availability” has been introduced, but we are not aware of any move to enforce it in LMICs.^2^, ^9^

Another major issue is that, when testing a new medicine, it is usual practice to enroll patients who have never used the medicine and are not receiving other therapeutic agents. In general, it may be better to test a new medicine without interference by other drugs: but is it ethical to conduct trials on patients who, for financial constraints or other reasons, are not receiving hydroxyurea, which has become the standard of care? One might argue that a medicine for SCD is valuable only when it offers something extra to patients who are already on hydroxyurea; not if it is just as good as hydroxyurea itself. Accordingly, unless there is a valid reason to the contrary, a new drug for SCD should be tested not against a placebo arm, but against a hydroxyurea arm.

Finally, we advocate that, if a trial is successful, there should be a compulsory 3‐year minimum of treatment access for study participants after trial completion in LMICs. This period would allow pharmaceutical companies and healthcare institutions to negotiate the conditions for providing subsequent supply and patient access.

### 1.3 Public health issues

In principle, clinical trials in LMICs can offer opportunities to address inequities related to geographical location, and socioeconomic status; and they may oppose racism. Industry‐sponsored multinational trials enable participants to access cutting‐edge treatments and to receive optimal standards of care with close monitoring of complications. At the same time, conducting clinical trials can strengthen the capacity of clinical centers, which benefits patients, healthcare systems, and also industry. From the point of view of the pharmaceutical industry, performing clinical trials in regions where SCD is highly prevalent can essentially set the scene for expanding market operations.^1^, ^8^, ^9^

**FIGURE 1:**
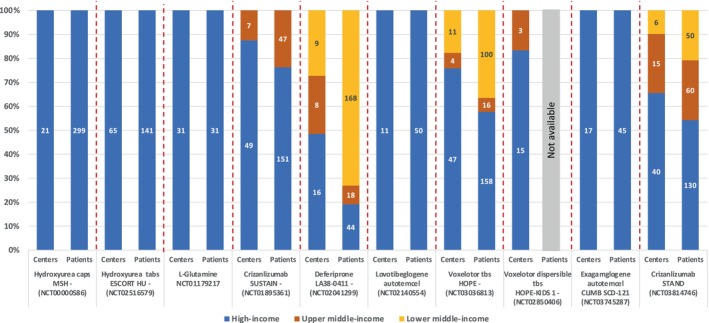
Distribution of centers and patient enrollment of pivotal clinical trials for sickle cell diseases by World Bank income classification criteria. [Color figure can be viewed at wileyonlinelibrary.com]

While hydroxyurea was tested and developed exclusively in North America and only subsequently investigated elsewhere, deferiprone, crizanlizumab, and voxelotor featured 10%–27% of enrolling centers from countries that are regarded as LMICs (Figure 1). In some cases, the enrollment of patients from LMICs was very significant. For example, in the SUSTAIN study of crizanlizumab, only 12% of centers were in LMICs, but they enrolled 24% of participants; in the STAND study, 34% of centers in LMICs enrolled 46% of participants. Similarly, a study of deferiprone featured centers from LMICs at 51% that enrolled 81% of participants, and for voxelotor the centers from LMICs accounted for 24%, but they enrolled 42% of the participants.

The promising advances in SCD therapeutics must be seen against the background of a situation whereby hydroxyurea, the global standard of care, is still under‐used in Africa. We recommend that providing hydroxyurea to all patients with SCD in Africa is a top priority.^10^ Indeed, the Model List for Essential Medicines of the World Health Organization—a technical guidance supporting countries' medicines selection based on cost‐effective criteria, and for international organizations to prioritize the procurement and supply of medicines—includes only hydroxyurea as a recommended treatment for SCD for both children and adults.^11^

## 2 MOVING THE FIELD FORWARD

The most innovative and exciting development listed in Table 1 is gene therapy: this has the promise of long‐term improvement and potentially curative intervention. Despite being licensed by stringent regulatory authorities, gene therapy should probably still be regarded as experimental, since long‐term follow‐up data are very limited and the vectors and techniques are still being improved. The price of these new products is more than $2 million to be paid upfront, which was claimed to be warranted by comparison to the astronomic prices of other newly approved treatments ($299 000 for L‐glutamine and $1.1 million for voxelotor and crizanlizumab over a lifetime).^12^ In principle, there is no reason why any curative or otherwise efficacious treatment should be denied to SCD patients in LMICs: however, in these countries, this price range is simply unrealistic and therefore cannot be considered a priority.

Beyond the specific research, ethical, and public health issues discussed above, we feel that the international community has a historical responsibility toward the global South and that in the immediate future, there should be a “pay‐back” at least to a small extent. Therefore, we propose that international organizations, policymakers, and the pharmaceutical industry come together to undertake a stepwise path.

First, hydroxyurea should be made available to all patients with SCD.^13^ In Africa, fixed‐dose treatment with hydroxyurea at 1000 mg/day has been estimated to be $16.5–54.6 per month at the retail market.^14^ For quantities provided by global vertical programs—as with HIV, malaria, and TB in the context of the Global Fund—the price could even be leveraged further, toward the recently proposed goal of $0.10 per 500 mg hydroxyurea capsule (i.e., around $ 6 per month at 1000 mg/day).^15^ If 1% of the pharmaceutical budget for SCD in the global North were invested in purchasing hydroxyurea, such a “global tithe” could be transformative for Africa.

Second, clinical research and regulatory strengthening ought to proceed hand in hand. So far, in Africa, the latter function has been carried out by individual National Medicines Regulatory Authorities, but there is a drive to establish an *African Medicines Agency* (AMA) that will be able to draw from the expertise available in African Universities, Research Institutes, and Teaching Hospitals. A sound regulatory structure will make African countries more attractive for pharmaceutical companies, which may be induced to place manufacturing on the continent. Regulatory harmonization could provide substantial benefits: African countries, by coming together, could negotiate more favorable conditions, based on the very fact that they will be representing a population of up to 1.2 billion people. At the same time, pharmaceutical companies would interact with a reliable multinational system (as already in place in the US and the EU), thus streamlining global marketing efforts.

In the meantime, African Authorities should implement a holistic approach to SCD, comprising education of health workers, network development, early diagnosis, local manufacturing of essential drugs, and timely therapeutic intervention, to keep a virtuous circle active through which SCD is clearly set as a priority in the public health agenda.

## 3 CONCLUDING REMARKS

Taken together, these data document a positive trend of drug development toward the global South, especially in those regions where SCD is most prevalent. When analyzing the number of enrolled participants, we confirmed that the shift is significant and will likely play an important role in strengthening the SCD ecosystem. This trend is encouraging but the shift of clinical trials for SCD toward LMICs is less evident in pivotal clinical trials, where centers from malaria‐endemic regions, particularly in Africa, are still underrepresented.

Expanding industry‐sponsored multinational clinical trials further into LMICs provides a great opportunity to improve clinical outcomes and local capacity.^2^ Expanding into LMICs from the initial phases of development of a new drug could help the fine‐tuning of late‐phase trials that include regulatory purposes. However, the exercise might be fruitless if not supported by subsequent measures to improve access to the population once a therapy has been found effective.

In the long term, the responsibility to the health of people lies with their respective governments, who will rightly put in place those measures deemed most appropriate to address the medical needs of their citizens. In the meantime, once again we appeal to the Global Fund^14^: in the trail of what they have accomplished for years with respect to malaria, tuberculosis, and HIV infection, which are primarily horizontally transmissible diseases, they might choose to include SCD, which is transmissible vertically, into a global program that will supply hydroxyurea to all patients with this condition. This request has been recently and forcefully requested by a highly qualified group of African hematologists.^16^

## CONFLICT OF INTEREST STATEMENT

The authors declare no conflicts of interest.

## Supporting information


**Data S1.** Appendix 1.

**Data S2.** Appendix 2.

## Data Availability

The data that support the findings of this study are available from the corresponding author upon reasonable request.
