# Supplementary figures and images for: Globalization in clinical drug development for sickle cell disease
Source: Am J Hematol. 2024 Nov 12;100(1):4–9. doi: 10.1002/ajh.27525 (PMC11625986; doi:10.1002/ajh.27525)

## Appendix 1

### Identification of studies via registers

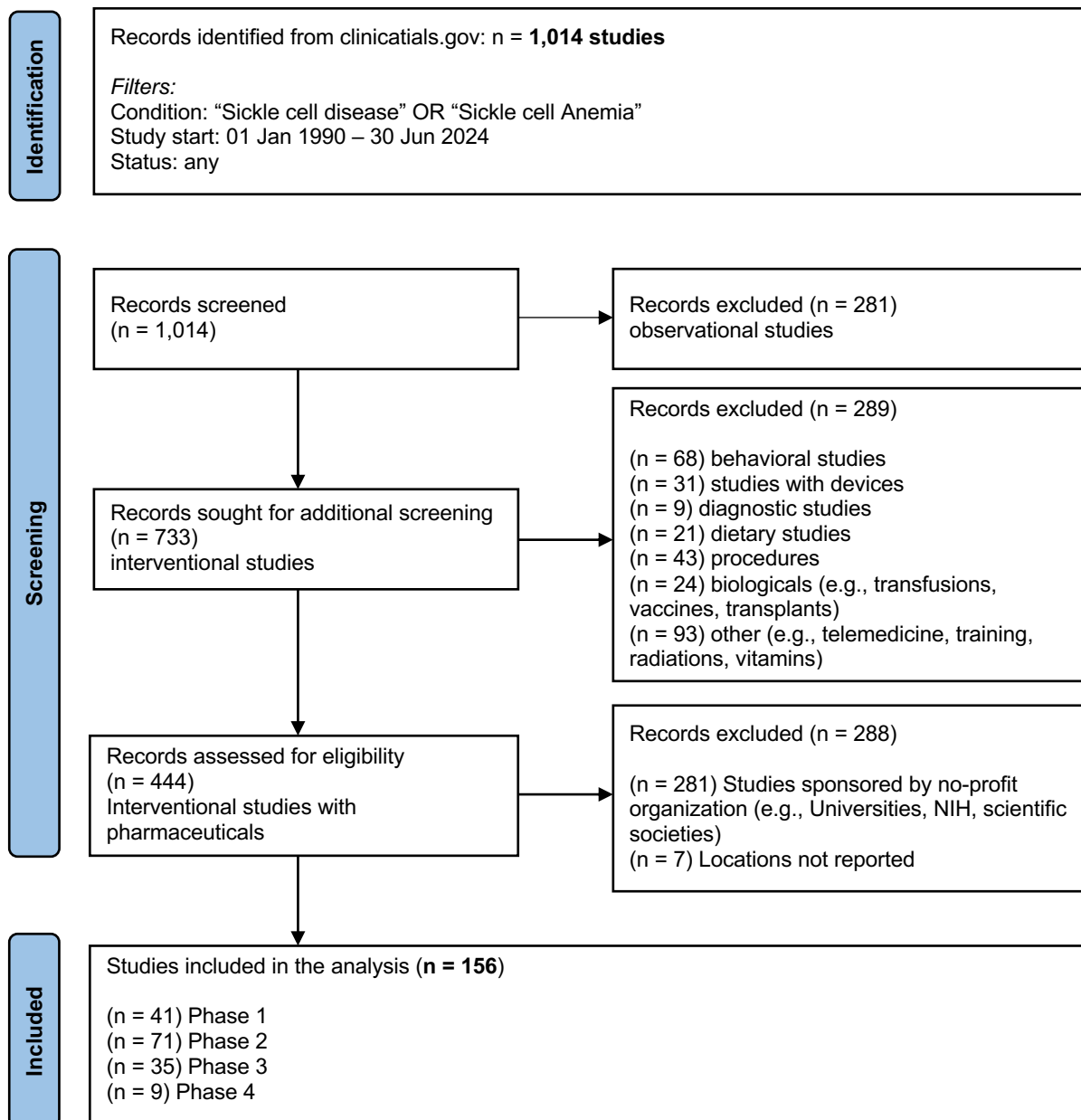

Supplement: Supplementary file 1 — Data S1. Appendix 1. [file AJH-100-4-s002.pdf]
