# Supplementary material for: Globalization in clinical drug development for sickle cell disease
Source: Am J Hematol. 2024 Nov 12;100(1):4–9. doi: 10.1002/ajh.27525 (PMC11625986; doi:10.1002/ajh.27525)

Appendix 2

A. Geographical distribution of centers concerning industry-sponsored Clinical Trials (CTs) for SCD stratified by phase of development

Regions

	Phase 1		Phase 2		Phase 3		Phase 4		Total	
	N	%	N	%	N	%	N	%	N	%
North America	150	69.4%	686	64.8%	894	51.2%	35	32.1%	1,765	56.4%
Europe	42	19.4%	175	16.5%	399	22.9%	24	22.0%	640	20.5%
Africa	6	2.8%	44	4.2%	185	10.6%	13	11.9%	248	7.9%
Latin America	8	3.7%	47	4.4%	113	6.5%	10	9.2%	178	5.7%
Asia & Middle East	6	2.8%	106	10.0%	147	8.4%	27	24.8%	286	9.1%
Australia	4	1.9%	0	-	7	0.4%	0	0.0%	11	0.4%
**Total**	**216**	**100%**	**1,058**	**100%**	**1,745**	**100%**	**109**	**100%**	**3,128**	**100%**

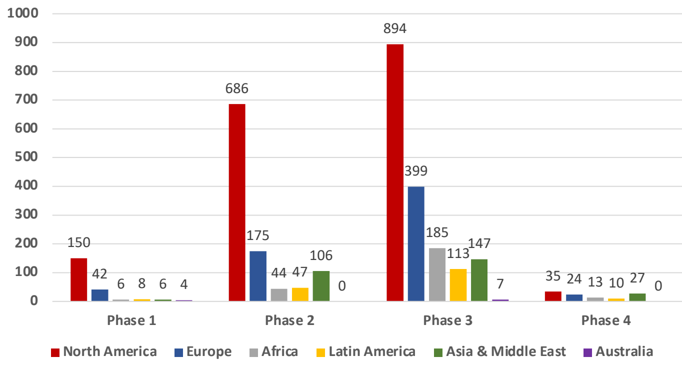

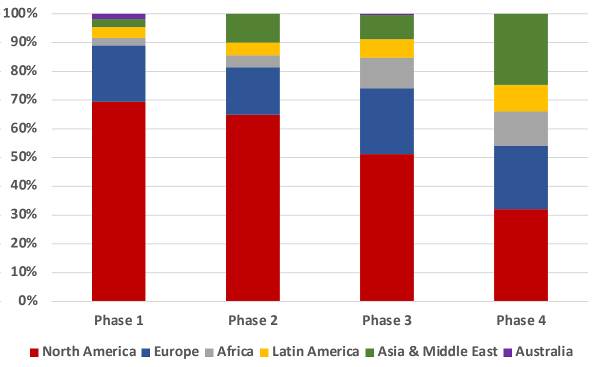

Income

	Phase 1		Phase 2		Phase 3		Phase 4		Total	
	N	%	N	%	N	%	N	%	N	%
High-income	200	92.6%	898	84.9%	1359	77.9%	64	58.7%	2521	80.6%
Upper middle- income	0	0.0%	58	5.5%	172	9.9%	23	21.1%	253	8.1%
Lower middle-income	16	7.4%	102	9.6%	214	12.3%	22	20.2%	354	11.3%
**Total**	**216**	**100%**	**1,058**	**100%**	**1,745**	**100%**	**109**	**100%**	**3,128**	**100%**

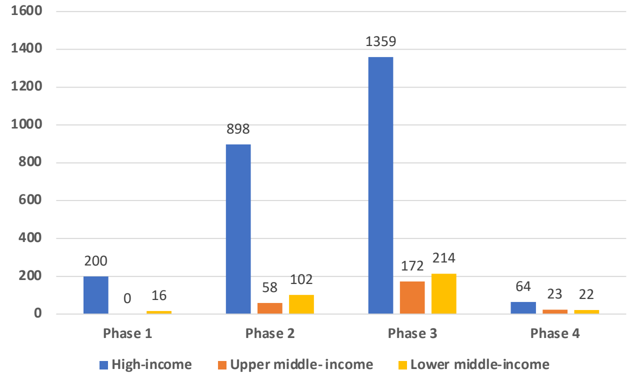

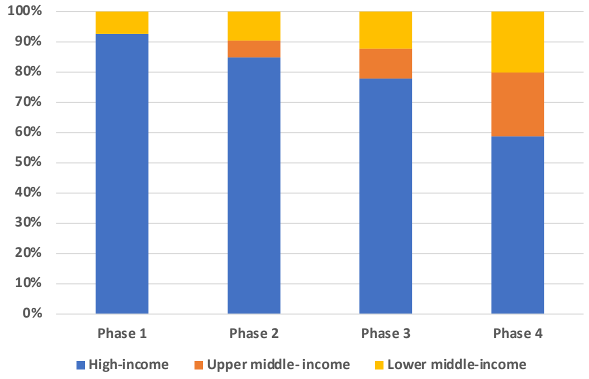

B. Geographical distribution of centers concerning industry-sponsored Clinical Trials (CTs) for SCD stratified by years of development

Regions

	2002-2006		2007-2012		2013-2018		2019-2024		Total	
	N	%	N	%	N	%	N	%	N	%
North America	399	63.1%	143	81.3%	658	63.5%	565	44.0%	1,765	56.4%
Europe	180	28.5%	12	6.8%	163	15.7%	285	22.2%	640	20.5%
Africa	3	0.5%	3	1.7%	73	7.0%	169	13.2%	248	7.9%
Latin America	7	1.1%	4	2.3%	52	5.0%	115	9.0%	178	5.7%
Asia & Middle East	36	5.7%	13	7.4%	90	8.7%	147	11.4%	286	9.1%
Australia	7	1.1%	1	0.6%	0	0.0%	3	0.2%	11	0.4%
**Total**	**632**	**100%**	**176**	**100%**	**1,036**	**100%**	**1,284**	**100%**	**3,128**	**100%**

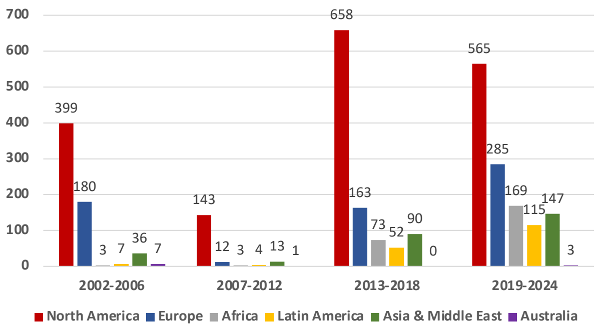

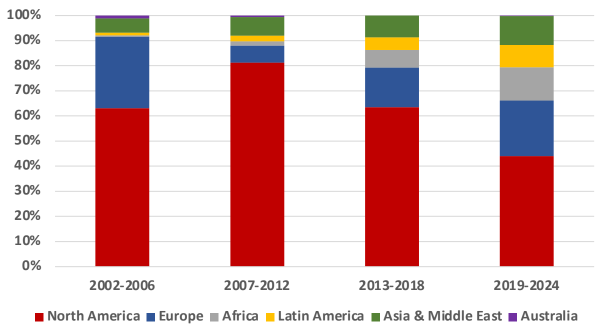

Income

	2002-2006		2007-2012		2013-2018		2019-2024		Total	
	N	%	N	%	N	%	N	%	N	%
High-income	611	96.7%	157	89.2%	859	82.9%	894	69.6%	2,521	80.6%
Upper middle- income	19	3.0%	4	2.3%	72	6.9%	158	12.3%	253	8.1%
Lower middle-income	2	0.3%	15	8.5%	105	10.1%	232	18.1%	354	11.3%
**Total**	**632**	**100%**	**176**	**100%**	**1,036**	**100%**	**1,284**	**100%**	**3,128**	**100%**

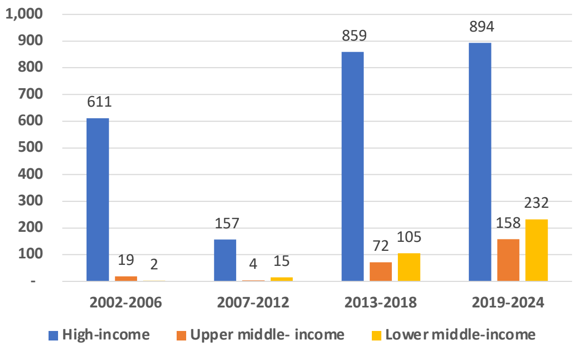

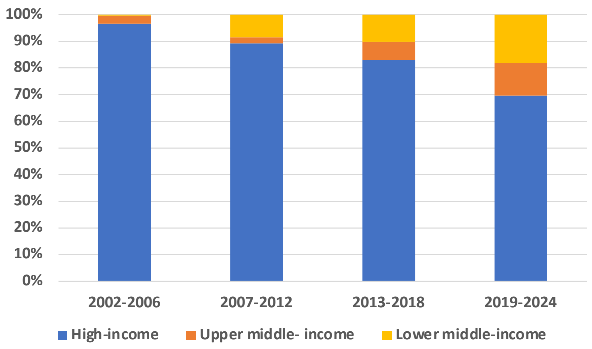

Supplement: Supplementary file 2 — Data S2. Appendix 2. [file AJH-100-4-s001.pdf]
